# Treatment challenges for urogenital and anorectal Chlamydia trachomatis

**DOI:** 10.1186/s12879-015-1030-9

**Published:** 2015-07-29

**Authors:** Fabian Yuh Shiong Kong, Jane Simone Hocking

**Affiliations:** Centre for Epidemiology and Biostatistics, Melbourne School of Population and Global Health, University of Melbourne, 3/207 Bouverie St, Melbourne, 3004 Australia

**Keywords:** Chlamydia, Treatment efficacy, Review

## Abstract

While true antimicrobial resistance to *Chlamydia trachomatis* is a rare occurrence, repeat chlamydia infections continue to be reported following treatment with a single 1 g dose of azithromycin or week long doxycycline – with considerable more concern about azithromycin treatment failure. While most repeat positive cases are likely to be reinfections, emerging evidence indicates treatment failure may play a role. Current data suggests that there may are differences in the efficacy of the drugs between rectal and non-rectal sites of infection and factors such as immune response, drug pharmacokinetics, organism load, auto-inoculation from rectum to cervix in women and the genital microbiome may play a role in treatment failure. Other possible reasons for repeat infection include the low discriminatory power of NAAT tests to differentiate between viable and nonviable organisms and failure to detect LGV infection. This review will present the current evidence regarding the management challenges for urogenital and anorectal chlamydia infections and provide some suggestions for where future research efforts are needed to address important knowledge gaps in this area and provide stronger evidence for the development of robust treatment guidelines.

## Introduction

In an era of increasing antimicrobial resistance, it is fortunate that *Chlamydia trachomatis* (CT) resistance remains rare [1]. However, there has been considerable recent concern about the efficacy of treatment for urogenital [2] and anorectal CT infections, [3] with particular worry about the efficacy of single dose azithromycin. Given that treatment failure could lead to ongoing CT transmission and an increased risk of complications associated with chlamydia, including HIV transmission, [4–6] it is vital that we understand the mechanisms of treatment failure and have access to highly efficacious treatments.

Concern about treatment failure has arisen because of high repeat CT infection rates observed in community cohorts of women in the UK (25.5 %) [7] and among women attending general practice clinics in Australia (22.3 %) [8] and the UK (29.9 %) [9]. Among men, repeat infection rates of up to 18.3 % have been reported for urethral infection [10] and up to 21.7 % for repeat rectal infections [11]. However, repeat infection does not necessarily mean treatment failure; repeat infection following treatment can also occur as a result of re-infection or it could be a false positive diagnosis due to the detection of non-viable (dead) chlamydia nucleic acid that is still clearing after treatment. Non-viable chlamydia nucleic acid can take about three weeks to clear after treatment [12].

The treatment guidelines for uncomplicated urogenital CT infections in the United States (US), [13] Europe [14] and Australia [15] all consistently recommend a single 1 g dose of azithromycin as the first line treatment. However the recommendations for the treatment of anorectal infections are less uniform with the US recommending single dose azithromycin while Europe and Australia recommending one week of doxycycline (100 mg twice daily) as first line therapy.

In this review, we will discuss the latest treatment efficacy data for anogenital chlamydia infection, examine the evidence about why treatment efficacy may vary between azithromycin and doxycycline and identify areas where further research is needed. We will refer to 1 gram single dose of azithromycin as “azithromycin” and 7 days doxycycline (100 mg twice per day) as “doxycycline” from hereon.

### Azithromycin and doxycycline efficacy for the treatment of anogenital chlamydia infection

A 2002 meta-analysis of randomised controlled trials (RCTs) examining the treatment of urogenital (cervical or urethral) chlamydia found no difference in efficacy between azithromycin (97 % efficacy) and doxycycline (98 %) (efficacy difference of 1.0 %; 95 % CI: -1.0 %, 2.0 %) [16]. However 11 of the 12 included studies used culture or immunoassays rather than sensitive Nucleic Acid Amplification Tests (NAAT) to assess microbial cure so it is possible that the efficacy estimates may have been overestimated [17]. Given such concerns and growing literature citing increasing reports of repeat positive infections, this meta-analysis was updated in 2014 [18]. The results of this analysis reported an overall efficacy of 97.4 % for doxycycline and 94.3 % for azithromycin (efficacy difference of 2.6 %; 95 % CI: 0.5 %, 4.7 %), suggesting a small, but statistically significant difference in favour of doxycycline. When this analysis was restricted to studies of symptomatic men only, there was a greater difference in efficacy in favour of doxycycline (efficacy difference of 5.5 %; 95 % CI: −1.4 %, 12.4 %). A recent meta-analysis of treatment efficacy for anorectal chlamydia infection found a much greater difference in efficacy: 99.6 % for doxycycline and 82.9 % for azithromycin (efficacy difference of 19.9 %; 95 % CI: 11.4 %, 28.3 %) [19].

Should we be alarmed at these results? For urogenital chlamydia treatment, no, we shouldn’t be alarmed. There was considerable variability in the quality of the studies included in the meta-analysis reducing the validity of their results. Firstly, only 17 % (4/23) of the trials included were double-blinded RCTs. Double blinding is necessary to ensure the risk of re-infection is similar between treatment arms because it is possible that taking a week long course of daily doxycycline may deter people from resuming sexual activity while taking treatment, making them less susceptible to re-infection. Secondly, most trials were based in high risk populations attending sexual health clinics. These populations are not representative of the majority of those who get chlamydia which is a largely asymptomatic infection.

However, for anorectal chlamydia infection we still don’t know which drug is the most efficacious. No RCTs comparing doxycycline and azithromycin were identified; the meta-analysis was based entirely on observational studies with 75 % (6/8) of the studies being retrospective case note reviews. Observational studies are at considerable risk of confounding and other biases that threaten the validity of their results. However, if azithromycin efficacy is indeed 83 %, then this is much lower than the 95 % threshold recommended by the World Health Organisation (WHO) for STI treatments and it shouldn’t be used for rectal chlamydia [20]. A treatment trial comparing azithromycin with doxycycline for the treatment of anorectal chlamydia infection is urgently needed to provide quality evidence to inform treatment guidelines.

### Antimicrobial resistance is unlikely to play a significant role in anogenital chlamydia treatment efficacy

To date, no prospective clinical studies have focused on the potential role of antibiotic resistance as a cause for chlamydia treatment failure. However, clinical treatment failures have been reported and the chlamydia isolates from these failures have been found to demonstrate multi-drug resistance *in vitro*, including resistance to tetracyclines (including doxycycline) and macrolides (including azithromycin) [21–26] - with mutations in a 23S rRNA gene been associated with *in vitro* resistance to macrolides [27, 28]. This resistance usually exhibits a heterotypic pattern where an infection has a small proportion of resistant organisms among a mostly susceptible population [1]. The phenomenon of heterotypic resistance has also been described in Staphylococcus spp. [29] and may evolve because of selective pressure from frequent exposure to antimicrobials [24, 30, 31]. This is further supported by *in vitro* demonstrations that chlamydia easily and rapidly develops resistance after serial passage in sub-inhibitory concentrations of macrolides [31]. To date, chlamydia strains exhibiting homotypic resistance in humans, a pattern where the whole population of organisms survive post treatment, have not been identified [1].

Chlamydia antimicrobial sensitivity testing is challenging, with few laboratories conducting it today. Minimum inhibitory concentrations (MICs) for chlamydia can vary depending on the cell line utilized and when the antimicrobial is added post infection [31]. There are few recent MIC data for chlamydia and as a result, it is not known whether there has been any “MIC creep” (decreased antimicrobial sensitivity) over time. However, given increasing concern about antimicrobial resistance for other STIs, it is imperative that we play closer attention to potential chlamydia resistance and collect chlamydia isolates from people who appear to have failed treatment for susceptibility testing.

### Organism load may be important for treatment efficacy

Heterotypic resistance is demonstrated *in vitro* at high levels of chlamydial organism load, but is not evident at lower levels of organism load leading to the hypothesis that treatment efficacy may reduce as organism load increases. A recent systematic review found that organism load is higher at the anorectal site than at cervical or urethral sites raising the possibility that anorectal infections may be more susceptible to treatment failure because of heterotypic resistance [32]. A recent Australian study investigating the association of organism load with repeat anorectal chlamydia infection among men, found that for every log_10_ increase in organism load, the odds of a repeat anorectal infection within 3 months of treatment with azithromycin increased by 70 % (OR 1.7; 95 % CI: 1.2–2.5) providing support for the hypothesis that high loads contribute to treatment failure [33].

The systematic review also found that those with symptomatic anogenital chlamydia infection have a higher organism load implying that those with symptomatic infection may be more likely to experience treatment failure [32]. The meta-analyses of urogenital treatment efficacy found that the efficacy for azithromycin was lower for those with symptomatic infection compared with doxycycline [18]. It is unclear why this is so and suggests that perhaps a longer duration of azithromycin may be needed [34] with animal studies suggesting chlamydia shedding was higher in those which were persistently infected and that extended courses can overcome persistent infections [35].

### Differences in the pharmacokinetic properties of azithromycin and doxycycline may have an impact on treatment efficacy

Doxycycline is highly lipid soluble which facilitates its rapid distribution into tissue and site of infection. On the other hand, azithromycin is delivered to the site of infection via phagocytic cells produced during the immune response to infection [36]. Data from animal studies suggest that, unlike urogenital sites, the immune response in the gastrointestinal tract is down-regulated so that chlamydia can continue to replicate and grow. If the innate immune response in humans is similarly down-regulated, then it is possible that there will be a reduction in phagocytes recruited to deliver azithromycin to the infection site. This is supported by mouse studies that have shown that chlamydiae resident in the gastrointestinal tract are not as susceptible to clearance by azithromycin as they are in the genital tract, [37] and a recent human study than found a dampened inflammatory response in the rectum in response to chlamydia [38]. This may explain in part the lower efficacy of azithromycin in rectal tissue compared with cervical tissue and the lower efficacy of azithromycin compared with doxycycline in rectal tissue. Nevertheless, pharmacokinetic data on the effective concentrations of azithromycin in rectal mucosa are urgently needed to determine whether a longer dosing regimen of azithromycin is needed for anorectal chlamydia infections.

### Persistent chlamydia infection may reduce treatment efficacy

Chlamydia persistence is another factor that might contribute to reduced treatment efficacy. CT, under the selective pressure of beta-lactam antibiotics, [39] interferon-gamma (IFN-Ƴ) or deprivation of nutrients such as iron and amino acids (e.g., tryptophan), can enter a persistent, metabolically inactive state containing enlarged reticulum bodies known as aberrant bodies (AB) [30, 40]. It is unclear how often the development of ABs occurs in vivo and whether it is due to either penicillin or IFN-Ƴ exposure, but ABs have been observed in *in vivo* samples from patients using electron microscopy [41]. *In vitro*, ABs are viable, but non-infectious and semi-refractory to treatment with azithromycin or doxycycline, depending on the cause of persistence. In this persistent state the organism can be detected by NAAT. A recent *in vitro* study examining the impact of β-lactam antibiotics on chlamydia persistence [39] found that all penicillins tested induced the formation of ABs with a 95 % reduction in chlamydia’s infectivity. Upon removal of the antibiotics, the chlamydia became infectious again, but β-lactam-induced persistent chlamydia was less susceptible to azithromycin *in vitro* [35]. Therefore, the question begs whether the marked increase in the use of beta-lactam antibiotics in recent years, [42] including its use in treating increases numbers of syphilis infections among gay men, [43] is contributing to antibiotic-induced persistence and whether increasing the duration of treatment can overcome this persistence [34] as has been demonstrated in animals [35].

IFN-Ƴ is generated as part of the innate immune response to chlamydia in humans and it triggers particular immune pathways which act to starve chlamydia of the essential amino acid tryptophan, leading to the development of ABs. In contrast to beta-lactam induced persistence, IFN-Ƴ exposure *in vitro*, makes chlamydia more resistant to doxycycline, but still susceptible to azithromycin [44].

Co-infection with herpes simplex virus can also contribute to persistence [45–48] while HIV co-infection does not [49]. Interestingly herpes co-infection does not mediate chlamydia persistence by any currently understood inducers, but through a novel mechanism that is yet to be fully understood.

Results from cohort studies examining the chlamydia isolates from those failing treatment among women [50] will provide useful insights in the possible reasons for treatment failure with similar studies needed with anorectal infections among MSM.

### The microbiome may play a role in treatment efficacy

Genital chlamydia has a unique interaction with their human host. The human response to infection (including chlamydia) is to produce IFN-Ƴ, which, among a host of pathways, upregulates the enzyme indoleamine 2,3-dioxygenase (IDO) which depletes tryptophan. The genital strains of chlamydia are tryptophan auxotrophs but have retained the trpBA genes in the tryptophan biosynthesis pathway. This enables them to back synthesise tryptophan from indole, a compound that can be present in the ano-genital tract as a product of some groups of bacteria (Eg: Prevotella, Fusobacterium, E. Coli) [51]. The availability of indole in the genital tract (the levels will vary depending on the composition of the microbiome), could rescue (i.e., recover or reactivate) chlamydia at this site from “attack” by the host [51, 52]. The balance of indole-producing bacteria in the genital microbiome could therefore influence whether an infection is acquired, is cleared or becomes persistent. Further research investigating the role of the microbiome on chlamydia acquisition and clearance will help us understand whether additional treatments such as probiotics or indole antagonists could reduce an individual’s susceptibility to infection, particularly re-infection.

### Treatment failure may actually be a false positive diagnosis

False positive diagnoses will happen if repeat testing takes place within 4 weeks after treatment. NAAT remains the recommended method for diagnosing CT infections [13, 53]. However, current NAAT tests are highly sensitive and do not differentiate between viable and non-viable (dead) chlamydia nucleic acid. Studies have shown that it is possible to detect chlamydia nucleic acid for about three weeks following treatment [12]. This is why length of time following treatment is an important factor for determining when to conduct a repeat test. Guidelines now recommend a “test for re-infection” at 3 months after treatment rather than a “test of cure” at 4 weeks post treatment to minimize the risk of a false positive diagnosis [13]. Further research is needed to develop new diagnostic tests that are able to quantify messenger RNA, a marker of viable, replicating organisms, rather than chlamydia DNA or ribosomal RNA, and use these new tests when re-testing people within 4 weeks after treatment.

### The misdiagnosis of lymphogranuloma venereum may reduce treatment efficacy

It is possible that in the absence of genotyping, cases of lymphogranuloma venereum (LGV) will be missed, leading to treatment failure because a longer 21 day regimen of doxycycline is recommended for the treatment of LGV [13]. There are several serovars of chlamydia based on the antigenic variations of the major outer membrane protein with serovars A-C associated with trachoma, D-K with urogenital, ocular and rectal infections and L1-L3 associated with a systemic infection called lymphogranuloma venereum [54]. LGV is usually managed on the basis of symptomatic clinical presentation, but there is now evidence that LGV can be asymptomatic. An audit of men attending an STI clinic in the Netherlands found that 27 % of rectal LGV cases were asymptomatic [55]. Other smaller studies in the UK and Germany have found between 17 % and 53 % of cases of rectal LGV among men were asymptomatic [56, 57]. These data suggest that rectal chlamydia infections among MSM should be genotyped to ensure LGV is diagnosed and treated appropriately to minimise the risk of treatment failure.

### Auto-inoculation of chlamydia from rectal to cervical site might contribute to treatment failure in women

There is increasing discussion in the literature about the potential role of auto-inoculation of cervical chlamydia infection from the rectal site. If rectal infection is indeed more difficult to treat with azithromycin than cervical infection, then auto-inoculation could contribute to repeat cervical infection in women [58–60]. Anal sex is increasing among heterosexual couples, with population-based data from the UK showing that that 15-17 % of heterosexual people reported anal sex in the last year, a 2–3 fold increase since 1990 [61]. There is also evidence that many women acquire rectal chlamydia infection in the absence of any reported anal sex [62].

A recent mathematical model estimated the impact auto-inoculation may have on azithromycin and doxycycline effectiveness for chlamydia in women and found that when the possibility of auto-inoculation is taken into account, doxycycline effectiveness is estimated to be about 97 % compared to just 82 % for azithromycin [63]. However, it is important to note that the efficacy estimates for treating rectal chlamydia included in the model were based on data from observational studies only and not from RCTs, reducing their validity.

Nevertheless, the available data suggest that we may need to consider collecting rectal swabs from women for chlamydia testing. However, rather than testing all women for both rectal and cervical infection which would increase testing costs substantially, consideration should be given to conducting rectal testing for women who present with repeat cervical chlamydia within three months of treatment and for high risk women who report anal sex. Further, consideration should be given to treating women presenting with repeat chlamydia with 7 days of doxycycline rather than 1 gram azithromycin.

### Treatment adherence may be important

It is important to note that azithromycin has definite advantages over doxycycline. It is single dose treatment, so non-adherence is minimized. Non-adherence with doxycycline can lead to treatment failure. In a secondary analysis of data from a RCT of men with non-gonococcal urethritis who were randomly allocated to either azithromycin or doxycycline, Khosropour and colleagues found that 28 % of men were non-adherent with their doxycycline (based on self-report). Among those men treated for chlamydia, those who were non-adherent had a nine fold increase in microbiological failure at follow up (RR = 9.3; 95 % CI: 1.0, 89.2) [64]. An earlier study which used Medication Event Monitoring System (MEMS) caps to monitor compliance, found that among 58 men and women who took at least 10 doses of doxycycline over 8 days, none (0 %; 95 % CI: 0 %, 6.1 %) failed microbiological cure compared with 20 % failure in those who took less than 10 doses (4/20; 95 % CI: 5.7 %, 43.3 %; *p* < 0.01) [65].

### Chlamydia screening and treatment could also be playing a role in higher repeat infection rates

As chlamydia is mainly asymptomatic, [54] regular screening of priority populations is considered a key public health control strategy. However there is ongoing debate of the potential negative effects of a ‘screen and treat’ policy. Partial immunity protecting against chlamydia re-infection has been demonstrated in animal models [66] with early antibiotic treatment impairing this protective immunity [67]. It has been suggested that while a ‘screen and treat’ strategy may reduce the incidence of chlamydia infection, it increases the risk of reinfection due to an impairment in the development of a partial immunity following treatment – this immunity occurring after a spontaneous resolution in the infection – the so called “arrested immunity hypothesis” [68]. Well-designed cohort studies of people at risk of chlamydia infection, with serial collection of genital specimens and samples for immunological investigation are needed to investigate this “arrested immunity” hypothesis in humans to determine whether treatment alters the immune response to infection.

## Conclusion

Our review has highlighted that there remain a number of gaps in our understanding about chlamydia treatment efficacy and that these gaps will continue to have implications for the clinical management of chlamydia infections; clinicians will continue to be concerned about the possibility of treatment failure in patients who present with repeat chlamydia infection. Although it is unlikely that antimicrobial resistance is an issue for chlamydia, formal mechanisms for the ongoing surveillance of chlamydia antimicrobial sensitivity should be established. While, most of these repeat infections will be due to re-infection, a small proportion may be false positive diagnoses because of retesting too early following treatment, and some will represent true treatment failure as a result of the mechanisms described above. The use of more discriminatory tests for detecting LGV and the development of tests to detect messenger RNA will improve clinical management of chlamydia.

Considerable gaps in the evidence about the most efficacious treatment for rectal chlamydia remain. RCTs comparing doxycycline and azithromycin are urgently needed but they must be must be double blind and placebo controlled to ensure that the risk of re-infection is similar between treatment arms; it is possible that taking a daily dose (as is required for doxycycline) may deter people from resuming sexual activity while taking treatment. Well-designed cohort studies of people at risk of chlamydia with serial genital-sampling will help determine the role of the immune response and genital microbiome in chlamydia acquisition and clearance and further our understanding about chlamydia persistence so that more efficacious treatments can be used. However, regardless of all the concern about azithromycin, we must be careful not to disregard this drug too prematurely based on the currently available data; azithromycin is a drug that can attain and sustain high tissue concentrations following a single dose with minimal issues with adherence and mild side effects, and it is effective for over 94 % of urogenital infections.

## Abbreviations

AB: Aberrant bodies
CT: Chlamydia trachomatis
HIV: Human immunodeficiency virus
IDO: Indoleamine 2,3-dioxygenase
IFN-Ƴ: Interferon gamma
LGV: Lymphogranuloma venereum
MEMS: Medication Event Monitoring System
MIC: Minimum inhibitory concentration
MSM: Men who have sex with men
NAAT: Nucleic Acid Amplification Tests
PCR: Polymerase chain reaction
rRNA: Ribosomal ribonucleic acid
RCT: Randomised controlled trials
STI: Sexually transmitted infections
WHO: World Health Organisation
